# Voluntary Hospital Finance

**Published:** 1923-07

**Authors:** Napier Burnett


### VOLUNTARY HOSPITAL FINANCE.

### A STUDY OF CONTRIBUTORY SCHEMES.

By SIR NAPIER BURNETT, K.B.E., M.D.

ONE of the most practically important papers read at the Conference was that by Sir N. Burnett on "Voluntary Hospital Finance," with especial reference to contributory schemes, with a suggested scheme for hospital treatment for "middle-class" patients. That Committees of Management might have the opportunity of full consideration of contributory schemes, he sent a *questionnaire* to 105 hospitals, the answers to which he analyses under a number of heads. It is obvious, he adds, that no hard and fast contributory method can be devised. Conditions vary too much with locality. At the same time, if the voluntary system is to be preserved, it is essential that the voluntary hospitals should have a well-thought-out policy, that is, a policy in its general features in consonance with the maintenance of a voluntary system.

### Existing Contributory Schemes.

The answer to the question, "How long has the contributory scheme been in force?" showed that 43 schemes have been in force less than 10 years; 13, 10 to 20 years; 8, 20 to 30 years; and 41, over 30 years. Sir Napier's comment upon these figures is that if payments by patients, or potential patients, are inimical to the voluntary system, a destructive process has been in progress at some hospitals for over 50 years. In 48 schemes the organisation is undertaken by the hospital; in 11 the organisation is undertaken by the local Hospital Saturday Fund; in 46 schemes the organisation is undertaken by outside committees. In more than 25 per cent. of these schemes there is no fixed amount of contribution; and the range of rates varies from a farthing to 3d. a week. Sir Napier continues :—

We may assume that a contributory scheme, as at present understood, aims at raising an amount of money that will pay the cost of treatment of contributory patients. The hospital has no desire to make a profit. Once profit is introduced there arises the vexed question of the remuneration of the honorary staff. Broadly, the position of the medical profession with regard to hospitals is that they will treat without payment the indigent. It may be assumed that they will continue to treat free those who have formed the great bulk of hospital patients for the last 50 years and of whom the majority form the present contributory class. It cannot be assumed that the medical profession will agree to any extension of this free service. It is now, therefore, a matter of importance for each hospital to be in a position to give the actual number of contributors treated and the amount of the money definitely attributable to its contributory scheme.

### The Contributor's Return.

In 74 cases the periodical payments entitle the contributor to free hospital treatment; in 31 they do not. Seventy schemes definitely undertake a liability, and 59 schemes cover the individual, his wife and children; 38 the contributor himself :—

If it be agreed that both the hospital board of management and the contributors themselves desire a contributory scheme to meet approximately the cost of treating contributory patients, then it must also be agreed that efforts should be made to arrive at such a weekly payment as will effect this object. The variations observed in the present rates and in the cover which they give show the uncertainty that prevails throughout the country. It is difficult to avoid the conclusion that the existence of some central body able to supply information and data would be a help to each hospital about to set up a new contributory scheme.

### The Voluntary Levy on Workers.

In 70 of the 105 cases there is no limitation of income. In 57 cases the voluntary levy is deducted from wages by the firm :—

It is an established fact that where the assistance of the employer can be enlisted in agreement with the employees, and the weekly payments deducted from the pay-sheet, that is, before the wages are paid, and the amounts sent in to the hospital direct, the total sum raised is almost invariably in excess of that produced by any other method. There are figures to show that this method has been instrumental in increasing threefold the amount raised at works and organisations previously dependent upon the good offices of some voluntary collector.

### The Employers' Contribution.

In 76 cases the employers do not contribute upon any recognised basis. The remainder vary. Some give from 25 to 200 per cent. of the men's contributions; £1 per £1,000 paid in wages; 5s. per 1,000 spindles (cotton spinners); 1s. a year for each contributory employé; and a penny or a halfpenny per week for each employé :—

Although employers are according facilities for the raising of money by allowing deductions on the weekly pay-sheet, there is yet room for increased effort in the direction of inducing them to make grants in proportion to the amount of the employees' contributions. In most districts firms are liberal subscribers to the funds. At the same time, an organised basis of support will on the whole and in the long run produce more money than spasmodic generosity.

### Co-relation of Work and Support.

Forty-four schemes do not attempt to co-relate support received with work done for an area, a district, or a contributing firm :—

There is no doubt that this co-relation of support and work done is one of the most powerful levers in appeal work. In one case in which the employés of a large firm were under the impression that the support they gave more than covered the cost, when as a matter of fact it fell short by several hundred pounds, the mere putting forward of the two figures resulted in a contribution almost sufficient to cover the difference. The actual number of contributors is a somewhat difficult figure to ascertain. At the same time, 10 have arrived at an approximation and realise its importance. No such difficulty, however, attaches to a record of the number of contributors treated, and the fact that 89 out of the 105 are not in possession of this figure shows that registration of patients in hospital is not keeping pace with the development of the contributory system.

### Inducements of a Contributory Scheme.

Sixty-five of the 105 schemes raised more in 1921 than in 1920, and 33 raised less; the remaining seven were started only in 1921 :—

The inducements to join a contributory scheme appear to be that by doing so a less fortunate comrade may be helped when in need of treatment; that the contributor and his family may get treatment should it be needed; that a contributor shares in the management of the hospital. If the voluntary system is to be maintained the emphasis should be laid mainly on the first. Contributory schemes differ from insurance in that they are appeals to the heart as well as to the head. There is widespread evidence of the desire to keep "the voluntary gift to help another" as the main idea in a contributory effort. The older schemes carefully set forth that contributors will be admitted if they are suffering from complaints suitable for hospital treatment, and if there is room in the hospital, in both cases the decision as to admission resting with the hon. staff of the hospital. In the newer schemes there appears to be a tendency to slur these points, or to disregard them altogether. Every argument is in favour of making the hospital liability clear from the outset.

### Financial Arrangements with Cottage Hospitals.

The apportionment of money raised by a contributory scheme among hospitals in an area is a matter of considerable importance, and also of some difficulty :—

The services rendered in the large hospital differ from those rendered in the small or the cottage hospital; the area which each hospital serves cannot be clearly defined, and the contributory schemes are run sometimes by the large hospital in the area; sometimes by some outside body, such as the Hospital Saturday Fund; sometimes by overlapping efforts on the part of each hospital. The difficulties connected with apportionment would appear to be less in those cases where the contributory scheme is controlled by some central body, either already existing, such as the Hospital Saturday Fund, or some other organisation specially set up for the purpose. There are, however, certain strong arguments in favour of each large hospital running its own scheme. Whether or not each small hospital should also run its own scheme is questionable—appeal work requires an organised staff and involves considerable expenditure. In those cases where a contributor to a scheme run by a large hospital has received treatment at (say, a neighbouring cottage hospital), that cottage hospital would appear to have a claim to a share of the contributory money. Should this principle be accepted, then the basis for apportionment would appear to be the work done by each hospital, wherever situated, for contributory patients. In one case of which we have heard, the main hospital has agreed with the neighbouring cottage hospitals upon definite areas of service, one-quarter of the total amount raised in each cottage hospital area being handed over to the cottage hospital. In another where the main hospital undertakes the whole organisation of the contributory scheme in an area, a definite rate of payment is made by the main hospital to each cottage hospital in respect of each contributory patient treated. This rate of payment is based, firstly, upon the relative value of the service rendered by the cottage hospital as compared with the main, and, secondly, upon the relationship that the total amount raised by the contributory scheme bears to the total cost of treating contributory patients in the area.

### HOSPITALS FOR THE MIDDLE CLASS.

When he comes to this very important subject, Sir Napier Burnett reminds us that in every community there exists an artificial classification of the people into upper, middle and lower, according to financial status. Much as we may dislike all such arbitrary divisions, yet the one suggested does exist, and for the purpose of the following remarks he accepts this division. For the sake of brevity these classes are referred to as "A," "B," and "C" groups.

### Conditions Ensuring Co-operation.

Two conditions stand out as worthy of the serious consideration of hospital committees, if contributory schemes are to ensure the co-operation of the various interested parties—namely :—

The fixing of an income limit; and a clear definition of the liability undertaken by the hospital, such as, hospital treatment to be offered, provided that: In the opinion of the medical staff the patient is a suitable case for hospital treatment; that hospital accommodation is available. These conditions being fulfilled, there would appear to be reasonable grounds for anticipating that the medical officers would continue to act in a voluntary capacity according to the original terms of their appointment on the staff of the hospitals.

### Plight of the Middle-class Patient.

Remarking that the plight of the "B" or middle-class patient when overtaken by accident or sickness requires no special pleading, Sir Napier enumerates the ways in which patients whose annual income exceeds £250 may at present receive hospital treatment :—

1.—A certain number find their way into the hospital wards, either as members of a contributory scheme which has no fixed income limit, or they are admitted as the result of some emergency illness, and while in hospital they may or may not be assessed towards the cost of maintenance by the hospital almoner. Certainly their presence in the hospital ward has been the occasion in some instances for a justifiable criticism on the part of the honorary medical staff. 2.—A comparatively small number of the larger hospitals has recently followed the lead given by many of the cottage hospitals in setting aside a small number of beds for paying patients. Such beds usually show a high percentage of occupation, indicating that they are meeting a real need. Patients admitted to those reserved beds are charged from two to five guineas per week towards the cost of maintenance, and in some hospitals the patient also pays for treatment, making his own financial arrangements with a member of the hospital medical staff. General hospitals in this country have not been designed, as in America, with the provision of separate accommodation for patients paying varying rates either towards maintenance or treatment, with the result that patients in adjoining beds occasionally find themselves paying different rates and consequently are prone to question the equity of the arrangements, whereas in other hospitals the medical staff are not expected to charge the patients in the paying wards for their services. Such an arrangement is likely to lead the medical officer to the opinion that his services are being exploited by the hospital in being asked to treat patients of a financial status considerably above those for whom his services were contracted for in a voluntary capacity. 3.—Again, a class "B" patient may be admitted to a general hospital as a member of some insurance scheme, such as the Sussex Provident Scheme, which undertakes to pay to the hospital the total cost of maintenance plus an agreed percentage to the medical staff for every member in the scheme treated by the hospital. But the maximum income limit of the Sussex scheme is fixed at £350 for husband, wife and family (up to sixteen years of age), and the annual premium for the provision of hospital treatment when required, plus dental and ambulance service, &c., is £2.

### A Voluntary Insurance Scheme.

Sir Napier looks forward to the time when a hospital bed will be available for every member of the community who needs it. Meanwhile the following scheme is submitted, not so much as a final solution, but as a contribution towards the solution of a difficult and urgent problem, namely, hospital provision for middle-class patients. "I suggest that, by a system of insurance, hospital provision might be arranged for the class 'B,' or middle-class, patient with an income, say, between the limits of £250 and £600 or £700 per annum, the premium for such insurance to be at the rate of £2 per annum with, the option of paying quarterly or half-yearly." By way of illustration, Sir Napier gives these figures :—

Say that in one community there are 5,000 people between the income limits above mentioned, who voluntarily join an insurance scheme in order to obtain hospital treatment when necessary, and when accommodation is available. On the basis of £2 per annum, the hospital would collect £10,000, which would be kept as a pool apart from the other hospital funds. The first call on the pool would be payment to the hospital of the total cost of maintenance of all insured patients treated during the year. The sickness incidence amongst class "C" is a known quantity—2 per cent. require hospital treatment. The sickness incidence in class "B" is considerably less, and also there is not the same liability to accident; but in order to provide a margin of safety, and also to include husband and wife, let us double the rate in "C" class and allow 4 per cent. of sickness incidence. Out of the 5,000 people insured, some 200 might require hospital treatment during the year. Further, if we calculate the average total cost to the hospital of each in-patient as £10—roughly, three guineas per week, and an average length of stay of three weeks—the hospital would receive £2,000 in full payment of the total cost of the insured patients treated. This payment would leave a balance of £8,000 in the pool.

### Limit of Voluntary Medical Service.

Under the contributory scheme system for the payment of class "C" patients, he suggests that hospitals have gone to the extreme limit in expecting medical men to give their services in a voluntary capacity :—

So that when a scheme of insurance for hospital treatment is suggested for patients belonging to the middle class even within the income limits mentioned, the medical staff could not reasonably be expected to render voluntary service. Therefore I suggest that the second charge on the pool should be payment to the medical staff; and in order to remove from the wards all possibility of varying tariff charges, an agreed percentage of the pool might be paid into a medical staff fund, to be apportioned by the medical men themselves to their individual members on the basis of work done. Again, to allow of a margin of safety, the maximum figure of 50 per cent. may be withdrawn from the pool towards such a fund—that is, a sum of £4,000. The further balance of £4,000 remaining in the pool after meeting the total charges incurred by the hospital, and the payment to the medical staff, represents the real insurance quota, or so-called profit of the scheme, and this sum should be utilised in the provision of increased accommodation and facilities for the treatment of class "B" patients.

### The Insurance Machinery.

Having dealt briefly with possible criticism,, one of which relates to the machinery of the scheme Sir Napier concludes :—

It has been suggested to me that the scheme might be carried out by some of the recognised insurance companies, who already possess the necessary collecting machinery. The obvious reply to such a suggestion is that the insurance company would undoubtedly seek to possess the remaining surplus as remuneration for running the scheme, and thus deprive the hospital of the outstanding benefit under the scheme. The requisite machinery might quite well be provided by the existing contributory scheme organisation. The three outstanding advantages that such a scheme, as roughly outlined, would possess are:—The elaborate equipment and facilities for the diagnosis and treatment of disease now possessed by the modern voluntary hospital and obtainable to the same extent nowhere else would be available for an additional and very deserving section of the community; a fresh avenue of finance would be opened up for voluntary hospitals; payment would be provided for the hospital medical staff.

---

### A Pamphlet on Insulin

Messrs. Burroughs, Wellcome publish a pamphlet on Insulin, with some account of its discovery, clinical evidence and a statement by the Medical Research Council as to the treatment of diabetes mellitus. The pamphlet, which is intended for medical men only, states that "already some of the limitations of Insulin have been defined. Its indiscriminate and improper use is likely to produce results which will give a wrong and harmful impression of its merits."

### PROBLEMS OF HOSPITAL ADMINISTRATION.

**By HERBERT L. EASON, C.B., C.M.G., M.D., M.S.**

In his opening sentences Mr. Eason said that he welcomed the opportunity of expressing his belief in the voluntary system.

### The Governing Body.

After some remarks upon the close corporation method of management as compared with the electoral body, he continued :—

However the main body of Governors of a Hospital is elected, the actual management is usually entrusted to a smaller Committee, which may be designated generally as the Committee of Management; and it is this Committee which should be elected with the greatest possible care, and should contain persons of experience and judgment, to the exclusion of the faddist and the person of narrow outlook. It is also very important to remember that a Voluntary Hospital is a charitable institution and not an insurance company for the mutual benefit of subscribers who will get out of the hospital the full value of all they put in. Hence in connection with contributory schemes for the financial assistance of hospitals there is a danger in the direct election of contributors' representatives to the governing bodies unless they clearly understand that they can never get their entire money's worth and that they are not only providing for their own benefits but also for the relief of that large community who by reason of sickness, poverty or unemployment, are unable to contribute even a small weekly sum.

### Representation of the Medical Staff.

Discussing the question whether the medical staff should have direct representation upon the Managing Committee, Mr. Eason said :—

Speaking for myself, being a member of the Medical Staff of my own hospital, I hold the view that it is not desirable that the medical staff should be directly represented on the Managing Committee, and for the following reasons: In the first place the Committee of Management is or should be the ultimate and sole authority in the hospital and in virtue of this authority should elect the medical staff, who therefore, whether they receive a nominal salary or none at all, are the servants of the Governors; if members of the medical staff sit upon the Committee of Management they are in the equivocal position of being simultaneously masters and servants. In the second place the discipline of a hospital must be in the hands of the Managing Committee and the Medical Staff obey such rules and regulations as are laid down from time to time by the Committee. It is not always desirable that when disciplinary action however slight has to be taken with regard to a member of the medical staff of a hospital that his colleagues should either know of it or be in a position to act as judges of his conduct. In the third place, the greater part of the work of a managing committee has no connection whatever with the professional or scientific side of the hospital's activities, and it is a waste of time for the medical staff to attend committees when their special knowledge is not required. I must, however, make it quite clear that it is of the utmost importance that the Medical Staff of a Hospital as a whole should be fully and frequently consulted on every matter connected with the professional aspect of a hospital's activities, in which the medical and surgical staff are concerned.

### A Joint Committee.

The best intermediary between the Governors of a hospital and its medical staff is a joint Committee meeting at frequent and regular intervals; on this Committee there should be representatives both of the Governors and of the staff :—

The Committee need have no executive powers and can be purely consultative; but its value lies in the fact that at its meetings all questions and grievances can be freely ventilated as between the staff and governors, and no official such as the Superintendent or Secretary can obstruct free intercourse between the professional and lay sides of the hospital. If it is understood that no question affecting the staff will ever be discussed, or at any rate decided at the Committee until it has been freely and fully discussed by the Medical Committee, the Governors will always be in possession of the considered view of their Medical Staff and will be able to avoid taking steps in the domestic or public policy of the hospital which will place themselves and their medical staff in a position of disagreement over matters of professional importance. It is over these questions of professional policy that most of the big difficulties arise in hospital management.

### The Superintendent or Secretary.

The Superintendent should be responsible for the good order and management of the internal affairs of the hospital, should take a general superintendence of all persons employed within the establishment, and should report to the Managing Committee any serious dereliction of duty on the part of anyone so employed :—

He should be the medium of communication between the authorities of the Hospital and other persons in all matters relating to his department. He should also be responsible for the admission and discharge of patients and for keeping the necessary records. Finally, he should take a general superintendence of the Hospital stores and articles required for consumption, and should supply the Managing Committee with regular summaries of articles used or consumed in Hospital. In carrying out the duties outlined in this last sentence it becomes his special duty to supervise the expenditure of the Hospital in all departments. The Superintendent should be, under the Managing Committee, the sole authority for all hospital expenditure.

### The Works Department.

Stress was laid upon the importance of a well-managed works department :—

If such a department is well run under a capable foreman of works not only can routine repairs be carried out more economically than by an outside contractor, but also many articles can be made instead of purchased, thus effecting the saving of outside profits. For example, in my own hospital not only does the works department include the necessary mechanics for plumbing, carpentering, bricklaying and painting, but also upholsterers, fitters, wiremen, coppersmiths, cutlers and metal platers. We make all our own mattresses, splints, operating tables, sterilisers, dressing tins, dressing tables, ward tables, and orthopædic apparatus. On the domestic side all the nurses' and domestic uniforms are made in our own sewing-rooms. The cost of articles thus made is far below that at which they can be bought, after allowing for the cost of labour, materials, and overhead charges. A works department, unless very strictly supervised and kept within reasonable limits, may become very costly, but if every man in it does a good day's work for a good day's wage it is a great saving to any large institution

### Nursing Salaries.

The improved pay of nurses, imperative as it had become, has seriously increased maintenance expenses :—

Nursing salaries have gone up materially since the war and, as far as I can see, will never come down again. The modern tendency is for the nurse to work shorter hours for higher wages. This development increases expenditure in many ways, for shorter hours mean more nurses, and a higher nursing salary list. More nurses mean increased nursing accommodation, more capital outlay for building, increased charges for nurses' maintenance, and an increase in the domestic staff. Again, an increase in the domestic staff means increased accommodation, and increased maintenance harges, and so the expenditure grows like a snowball.

c

### Medical Salaries.

Medical salaries are also becoming an increasing charge upon the hospital finances :—

I think it should be made clear to the subscribing public that this large salary list is not due to the remuneration of the visiting staff. They are usually entirely honorary or paid a mere token remuneration, usually an amount settled one or two hundred year ago. The large medical salary list is due to the increasing number of junior medical officers who are required for the efficient service of a hospital ; and they are no longer content with the low salaries which were considered adequate a few years ago. The increase in the cost of living and the depreciation in the purchasing power of money has made it incumbent upon the governing bodies of the hospital to pay increased honoraria to registrars and departmental officers. In fact, medical salaries have increased in nearly the same proportion and for the same reasons as have mechanics' wages, and there is little hope of their ever returning to pre-war levels.

### Supervision of Consumption.

The secret of economy in hospital expenditure lies in the careful supervision of consumption :—

Returns should be made in respect of dietary, groceries, brooms, brushes, and cleaning materials, hardware and dressings. Each return should be drafted to show in each ward or department the actual quantities and the cost of every article ordered by the sister or head of the department. In hospitals where wards vary in size or in character some allowance must be made for the fact that some wards cost more than others. In order to make due allowance for the size of the ward, and the average number of patients in daily residence, the cost or quantities may be reduced to a common unit—for instance, that suggested recently by King Edward's Hospital Fund, 100 patient days, and I find that sisters of wards have no difficulty in appreciating the significance of such a unit. It has been my own experience to inherit such a system from my predecessor, Sir Cooper Perry, who introduced such forms at Guy's over fifteen years ago, and I can say without the slightest hesitation that these forms, which include every article ordered by a sister, are read and considered by the sisters with the greatest interest. Not only does such a system result in an actual saving in articles consumed, but every sister, from the moment she is put in a position of authority, realises that her duty is to economise as far as possible in the interests of the hospital, and that she is in friendly rivalry with her colleagues to achieve this end.

### The Matron.

What should be the position and duties of the matron ?—

Equal on the nursing side with the superintendent on the administrative, the matron should have a very real authority and responsibility in her department of the hospital. She is, of course, responsible for the engagement of nurses, their education and their discipline, and for making recommendations for their promotion either to wards or administrative posts. In my opinion, however, in addition to these duties, she should be responsible for the purchase of such domestic articles as bedding and linen and for the supervision of the hospital kitchens. The matron, if she has been properly trained, can supervise the hospital kitchen in quite as effectual a way as a steward, and the association of the feeding of the nurses and patients with the actual administration of the nursing and domestic staff makes her position much more logical and complete. Training as a kitchen sister under a capable matron makes an admirable preliminary education for any woman who herself aspires to become a matron. The matron must also frequently visit the wards, sculleries, and sanitary blocks of the hospital and see that they are kept in good condition, and she should carefully note whether directions affecting in-patients in regard to diet, nursing, personal cleanliness, &c., are duly carried out by the nursing staff. It may be said that these duties are too onerous for any one woman, and that either she should be relieved of some of them or have the assistance of advisory committees, such as Education and Nursing Committees. My own view is that a competent matron can carry out such duties, and that one efficient woman at the head of affairs makes an atmosphere and sets a standard far better than any committee.

### The Supply of Nurses.

One of the acute problems of the time is the maintenance of the supply of nurses :—

The present supply of probationers is most precarious, and there are few hospitals that have a full nursing staff. This scarcity of nurses has been most acute since the war, and is probably due to the war itself, as many careers have now arisen which afford occupation for women, are as well paid as nursing, and afford more opportunity for amusement and leisure. Before the war probationers were not usually admitted for training until they were twenty-three years of age or over. Efforts to enlist the sympathy of head mistresses in bringing nursing as a career to the notice of girls at school are met by the rejoinder that it is useless to try to get girls to take up nursing as a career unless the training can commence immediately after the school training ceases. If there is a compulsory interval between a girl leaving school and commencing her training, she drifts off into some other occupation or career and the school mistresses can be of no assistance. Probationers are therefore admitted into many hospitals now at the age of 19, but the experiment is far from being a success. Girls of this age very often do not know their own mind, and after a course of instruction in a preliminary training school many of them come to the conclusion that nursing does not appeal to them, or that it is too hard, or after successfully completing their preliminary training, and with a few days' experience of the wards, they come to the matron saying that a hospital is a horrible place and that they must go home that very afternoon. The percentage of sickness among young nurses is also much higher than among older women, as they are not sufficiently resistant to disease. It therefore happens that of every batch of probationers entering a preliminary training school at a hospital, from 25 to 50 per cent. may leave the hospital before the completion of their first year of training.

### Tightening the Curriculum.

Increasing educational demands help to make the probationer's life more strenuous :—

The modern tendency is for the training of nurses to become more and more advanced on the scientific side, so that there appears to be a danger that the curriculum for probationers will be little less than that of the woman doctor. As most of this mental work has to be done by the nurse in her off-duty hours, when she is usually physically fatigued, the strain may soon become intolerable. As it is essential that a nurse should have a good physique, and physique and intellectual capacity do not always go hand in hand, there may be a danger that, in demanding too high an intellectual standard from our probationers, we shall lose those women who are by nature physically best fitted to undertake the strenuous life of the hospital nurse. Nursing in the wards is also becoming harder and harder every day owing to the increasing demands of medical service. Investigations are becoming so complex, bismuth meals and special diets are adding so greatly to the routine work of a ward, and the number of persons who now have duties in the wards and require the attention of the nursing staff is increasing to such an extent that it is perfectly impossible to staff a hospital with the number of nurses that was adequate a few years ago.

### Nurses' Pensions "The Problem of the Hour."

For the trained nurse an adequate pension scheme is the problem of the hour :—

The pensions to which nurses can aspire at the end of their professional life are (says Dr. Eason) in most cases miserably inadequate, and a nurse is usually worn out at fifty. Her professional career is very short, and unless she is of a rigidly economical frame of mind, the end of her life may be a sad struggle with actual poverty. It is essential, therefore, in order to attract nurses of the right type in sufficient numbers to train at our hospitals, that every attempt should be made to make their life, while in training, attractive by the provision of comfortable bedrooms, good sitting rooms and amusement in the way of week-end cottages, sports grounds, nurses' leagues and all forms of co-operative interest and social enjoyment.

### The Almoner.

Mr. Eason thinks that in their social work the English hospitals are still "considerably behind" those of the United States, notwithstanding the progress that has been made in recent years :—

The almoner (Dr. Eason went on to say) should be the "servant" of the Governors and not partly or wholly the superintendent of the hospital. Payments from Samaritan funds should not be made by her, but by the governing body of the hospital or its representative, the superintendent, upon her recommendation. This procedure is consistent with the general principle that one person should be responsible for all the expenditure of the hospital. The position of the almoner under such a system is in no way derogatory to her, for if her work is sound, her recommendations are practically always accepted. And rightly so, for the almoner is one of the most valuable of the hospital's servants. She is in most cases, at any rate in a training school for nurses and students, the only permanent official dealing with patients. Nurses and doctors come and go, but the almoner remains, and the medical staff are therefore often dependent upon her for records of old patients, reports of home conditions and for information as to many unwritten regulations and customs. An efficient hospital almoner will reduce rather than increase hospital expenditure by means of systematic inquiries and co-operation with outside bodies.

### An Out-patient Inquiry Officer.

Mr. Eason concluded by advancing a proposition which he thinks may not be generally accepted by hospital experts :—

The duties of the almoner should not include that of inquiring into the circumstances of patients in order to ascertain whether they are eligible for treatment at a hospital. This is the duty of an out-patient inquiry officer, who in the capacity of a censor represents the governing body and is acquainted with the income limits which have been laid down to govern the admission of patients. The office of the almoner is to assist patients who have been accepted for treatment. If with that duty is combined that of inquiry officer, patients may come to look upon the almoner's department with some hostility, and its efforts to assist may be prejudiced.

### HOSPITAL FINANCE IN ITS RELATION TO APPROVED SOCIETIES

By VISCOUNT HAMBLEDEN.

THE title which has been chosen for this paper might suggest that the finance of Voluntary Hospitals has been greatly affected by the grant which some of the Approved Societies have agreed to make in respect of certain of their members who are treated as in-patients. The figures which I shall presently give you will show that although the amount received has been considerable and tends to increase, it does not at present form more than a fraction of the sums received in payment by or on account of patients. In principle, however, the payment is an important and welcome departure from the policy followed by Approved Societies up to the date of the last valuation. The societies having decided in principle to set aside a definite sum, it was necessary to devise means by which the amount available could be most equitably divided Donations pure and simple were ruled out, inasmuch as they did not satisfy the requirements of the Ministry of Health.

### Basis of the Scheme.

The basis of the scheme so far as the two great industrial societies were concerned is a payment in the first instance of £1 1s. per week in respect of each member entitled to additional benefits—that is, those members who were insured not later than December, 1918, and were still members of the society on July 5, 1920. The National Insurance Benefit Society administers benefits both out of realised surplus and out of benefit funds under Section 21, but the number of patients fron that society for whom payment has been made is comparatively small. So far as I know there have been no serious difficulties in the London area. The Prudential paid out in 1922 £83,000, or little more than half the amount of £150,000 set aside, for 45,000 weeks' treatment. The National Amalgamated paid out £42,138 for 24,072 weeks' treatment. The National Deposit Friendly Society paid out £10,224 for the treatment of 2,234 persons, or less than half the sum expected by them, as will be seen if we divide the total of £75,000 into five portions. The other two societies paid out less than £1,000 between them. The total paid amounts to over £135,000, a sum by no means to be despised. One interesting point in connection with these payments is the fact that the amounts are much greater in the later than in the earlier quarters of the year. From these figures it would appear that hospitals were somewhat slow to avail themselves of the grant, for it does not seem probable that more insured persons were treated in the last than in the first quarter.

### Some Hospital Figures.

Now turn to some figures obtained from hospitals. I have not attempted to get returns from all the hospitals in the country, but I have received figures from more than 100 general hospitals of over 100 beds. From these returns it appears that in London General Hospitals insured persons are more than 20 per cent. of all in-patients, who numbered 97,761, but that only 9 per cent. of all patients were qualified for the additional benefit under the scheme. The variations in the figures are remarkable, for one hospital returns only 1 per cent., and another less than 1 per cent., although in the latter case all insured persons taken as in-patients numbered 17 per cent. of the whole.

### Proportion of Insured Patients.

I can hardly think that in this case and some other cases full advantage has been taken of the grant. Some of the special hospitals show a much higher average. A chest hospital has received grants for 30 per cent. of all its patients, but an ophthalmic hospital for 8 per cent. only, and an orthopædic hospital for only exactly the same number. The amount received by the London hospitals from which I have received returns was £41,332, on account of 9,915 patients. The striking feature in the figures which I have obtained from provincial hospitals is the much higher percentage of insured persons treated by them. In the 35 hospitals in England which were able to give me complete returns, of 108,000 patients 33,828 were insured persons, or over 31 per cent. Scotland shows a still higher average, for of the 34,000 patients of whom I have records 13,200 were insured, or 40 per cent. In England, outside London, the proportion of patients for whom some payment was made was 87 per cent. of all in-patients, and the total amount received was £57,498.

### Two Criticisms.

It must not be thought that I am ungrateful for the undoubted help which this grant from the Approved Societies has brought to hospitals if I mention two main criticisms. In the first place, the basis of the scheme is not one which makes for permanency. The grant is made out of realised surplus, and if it is to depend upon the realisation of a surplus in the future its very basis is precarious. In the second place, some Societies may have surpluses, others may have none, and in the case of those fortunate enough to have balances to dispose of, their disposition depends upon the votes of their members. In the existing scheme, Societies representing only a little more than half the insured persons in the country are taking part; in the case of the remainder, when a surplus existed it was decided to use it for additional cash benefits. It cannot, therefore, be safely asserted that the scheme in its present form is of a permanent nature; that is to say, is certain to be continued after the next valuation. We may be called upon a few years hence to face the very important questions which must necessarily arise if this scheme is withdrawn and another, based not only on realised surplus, but on income also, is suggested.

### The Future.

We may, in fact, have to decide whether voluntary hospitals can take a definite and permanent part in the scheme of National Health Insurance. In the course of conversations with those who conduct the business of these Societies, I have been struck by the real interest which they take in the question of National Health, and I think this interest may lead not only to improvements in the panel system, but to a desire to carry that system a step further. Are we prepared to explore more completely the path upon which we have already set out? I am the less afraid to follow that course because I believe the voluntary system is in a stronger position to-day than it was three years ago. It would be a great mistake to give any encouragement to the idea that those responsible for the management of hospitals are prepared to surrender their principles for a cash payment. But the very fact that those principles are so strongly held and so widely supported should encourage us to preserve an open mind upon any scheme which can be proved to bring us nearer to a more perfect system of curing the sick and alleviating the troubles of the suffering.

